# Oral Preconditioning of Donors After Brain Death With Calcineurin Inhibitors vs. Inhibitors of Mammalian Target for Rapamycin in Pig Kidney Transplantation

**DOI:** 10.3389/fimmu.2020.01222

**Published:** 2020-06-18

**Authors:** Sepehr Abbasi Dezfouli, Mohammadsadegh Nikdad, Omid Ghamarnejad, Elias Khajeh, Alireza Arefidoust, Sara Mohammadi, Ali Majlesara, Mohammadsadegh Sabagh, Negin Gharabaghi, Modar Kentar, Alexander Younsi, Christoph Eckert, Tanja Poth, Mohammad Golriz, Arianeb Mehrabi, Arash Nickkholgh

**Affiliations:** ^1^Department of General, Abdominal and Transplant Surgery, Ruprecht-Karls University, Heidelberg, Germany; ^2^Department of Neurosurgery, Ruprecht-Karls University, Heidelberg, Germany; ^3^Institute of Pathology, Ruprecht-Karls University, Heidelberg, Germany

**Keywords:** oral preconditioning, brain death donor, kidney transplantation, pig, calcineurin inhibitors, inhibitors of mammalian target for rapamycin, TNF-α

## Abstract

**Background:** The systemic inflammatory cascade triggered in donors after brain death enhances the ischemia-reperfusion injury after organ transplantation. Intravenous steroids are routinely used in the intensive care units for the donor preconditioning. Immunosuppressive medications could be potentially used for this purpose as well. Data regarding donor preconditioning with calcineurin inhibitors or inhibitors of mammalian target for Rapamycin is limited. The aim of this project is to investigate the effects of (oral) donor preconditioning with a calcineurin inhibitor (Cyclosporine) vs. an inhibitor of mammalian target for Rapamycin (Everolimus) compared to the conventional administration of steroid in the setting of donation after brain death in porcine renal transplantation.

**Methods:** Six hours after the induction of brain death, German landrace donor pigs (33.2 ± 3.9 kg) were randomly preconditioned with either Cyclosporine (*n* = 9) or Everolimus (*n* = 9) administered via nasogastric tube with a repeated dose just before organ procurement. Control donors received intravenous Methylprednisolone (*n* = 8). Kidneys were procured, cold-stored in Histidine-Tryptophane-Ketoglutarate solution at 4°C and transplanted in nephrectomized recipients after a mean cold ischemia time of 18 h. No post-transplant immunosuppression was given to avoid confounding bias. Blood samples were obtained at 4 h post reperfusion and daily until postoperative day 5 for complete blood count, blood urea nitrogen, creatinine, and electrolytes. Graft protocol biopsies were performed 4 h after reperfusion to assess early histological and immunohistochemical changes.

**Results:** There was no difference in the hemodynamic parameters, hemoglobin/hematocrit and electrolytes between the groups. Serum blood urea nitrogen and creatinine peaked on postoperative day 1 in all groups and went back to the preoperative levels at the conclusion of the study on postoperative day 5. Histological assessment of the kidney grafts revealed no significant differences between the groups. TNF-α expression was significantly lower in the study groups compared with Methylprednisolone group (*p* = 0.01) Immunohistochemistry staining for cytochrome c showed no difference between the groups.

**Conclusion:** Oral preconditioning with Cyclosporine or Everolimus is feasible in donation after brain death pig kidney transplantation and reduces the expression of TNF-α. Future studies are needed to further delineate the role of oral donor preconditioning against ischemia-reperfusion injury.

## Introduction

During the process of organ transplantation, the ischemia reperfusion injury (IRI) together with the systemic inflammatory response to brain death causes infrastructural organ injury which could lead to initial poor function and ultimately primary non-function (1). The increased intracranial pressure and the absence of cerebral flow during brain death activates a full-blown neuronal, hemomdynamic and hormonal storm. The consequence is an inflammatory cascade which releases proinflamatory cytokines, chemokines and adhesion molecules and leads to infiltration of T-lymphocytes and macrophages in the organs (2). It has been shown that the treatment with methylprednisolone in donors after brain death (DBD) exerts protective effects against IRI in terms of decreased incidence of acute rejection (3).

Preconditioning with calcineurin inhibitors (CNIs) has been shown to have protective effects in a model of renal transplantation in rats compared to vehicle-treated animals (4). This renoprotective effect was seen with only one dose of CNI and was not different between cyclosporine and tacrolimus regarding measured outcomes. To the best of our knowledge, there is no data available regarding the donor preconditioning with CNI in a big animal (porcine) model. Everolimus (Certican), an inhibitor of mammalian target for Rapamycin (mTORi), inhibits the proliferation and the clonal expansion of antigen-activated T-cells, making it an interesting candidate for the pharmacologic preconditioning against IRI in the setting of DBD. Currently, there is very few data in the literature regarding this possible protective effect of Everolimus.

The aim of this study has been to investigate the feasibility and the effects of oral preconditioning of DBD donors with CNI (Cyclosporine A) vs. mTORi (Everolimus) vs. conventional administration of steroid in a porcine model of kidney transplantation.

## Methods

German landrace pigs (weight: 33.2 ± 3.9 kg) were given access to standard laboratory chow (ssniff R/M-H, ssniff Spezialdiäten, Soest, Germany) and tap water before experiments. The study protocol was reviewed and approved by the responsible animal welfare state authority (Regierungspräsidium Karlsruhe, Baden-Württemberg, Germany (file number: 35-9185.81/G-5/16) and were performed according to the institutional guidelines at the Ruprecht-Karls Univesity, Heidelberg, Germany in accordance with the guidelines of FELASA (Federation for Laboratory Animal Science Associations).

### Experimental Design

All operations and investigations were performed under general anesthesia. After premedication (azaperone 6 mg/kg intramuscularly (i.m.), ketamine 10 mg/kg i.m., and midazolamine hydrochloride 0.5 mg/kg i.m.), anesthesia was induced with ketamine [1 mg/kg intravenously (i.v.)], midazolamine hydrochloride (0.1 mg/kg i.v.), and atropine (0.04 mg/kg i.v.). During the operation, anesthesia was maintained with 1.5–2% isoflurane. Ventilation was pressure-controlled in a half-closed system. The ventilation parameters included a tidal volume of 240 ml, frequency of 17/min, maximum pressure of 24 cmH_2_O and positive end-expiratory pressure of 3–5 cmH_2_O. The pH, HCO_3_, pCO_2_, and pO_2_ concentrations were determined by routine analysis of arterial blood gases. The respiration parameters were then adapted to these values. During surgery, controlled infusion therapy was applied using 20 ml/kg/h Sterofundin (B. Braun, Melsungen, Germany). The mean arterial pressure (MAP) as well as the central venous pressure (CVP) and the heart rate (HR) were continuously monitored. For these reasons, the right common carotid artery and the jugular vein were first prepared, cannulated and connected to pressure transducers. The central venous catheter additionally served for volume substitution, for the administration of pharmaceuticals and for obtaining central venous blood samples.

### Induction of Brain Death

Our standardized method for the induction of brain death in pigs has been published elsewhere (5, 6). Briefly, under general anesthesia, two burr holes (diameter, 10 mm) were placed epidurally in the left temporal (CODMAN® MICROSENSOR® Integra LifeSciences, Plainsboro, NJ, USA), right temporal (10-French Tiemann balloon catheter, B. Braun, Melsungen, Germany), and intraparenchymal in left frontal (thermal diffusion probe), regions. The slow inflation of the epidurally inserted Tiemann balloon catheter with a total of 6–13 mL NaCl 0.9% solution (running rate: 1 mL in 3 min) caused brain death within about 60 min. Brain death was confirmed after cessation of anesthesia by (1) the typical hemodynamic changes of brain death, (2) the absence of response to painful stimuli, and (3) the absence of pupillary and corneal reflexes. Ventilation and close monitoring of cardiovascular parameters such as heart rate and blood pressure were continued during organ procurement.

### Preconditioning of Donor Animals

**Figure 1:**
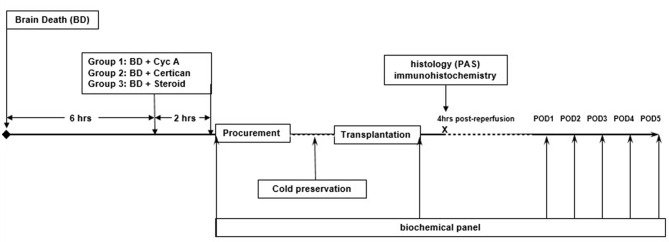
Study design. Six hours after the induction of brain death, German landrace donor pigs (33.2 ± 3.9 kg) were randomly preconditioned with either Cyclosporine (*n* = 9) or Everolimus (*n* = 9) administered via nasogastric tube with a repeated dose just before organ procurement. Control donors received intravenous (i.v.) Methylprednisolone (*n* = 8). Kidneys were procured, cold-stored in HTK solution at 4°C and transplanted in nephrectomized recipients after a mean cold ischemia time of 19.32 ± 2.92 (SD) hours. No post-transplant immunosuppression was given to avoid confounding bias. Blood samples were obtained at 4 h post reperfusion and daily until postoperative day (POD) 5 for complete blood count, blood urea nitrogen (BUN), creatinine (Cr), and electrolytes. Graft protocol biopsies were performed 4 h after reperfusion to assess early histological and immunohistochemical changes.

Six hours the after induction of brain death (i.e., 2 h prior to organ procurement) preconditioning was performed with the oral administration of Cyclosporine suspension (Novartis Pharma GmbH, Nuremberg, Germany) (10 mg/kg body weight) (*n* = 9) or Certican suspension (2 mg) (*n* = 9) (Novartis Pharma GmbH, Nuremberg, Germany) - via the nasogastric tube. Doses were analogous to usual administered doses in adult organ transplantation. A repeated dose was administered immediately before organ procurement. Control group (*n* = 8) received 250 mg intravenous bolus of Methylprednisolone (Urbason®, SANOFI-AVENTIS GmbH, Vienna, Austria) then continuously at a dose of 100 mg/h until procurement (Figure 1).

### Organ Procurement and Preservation

A full-length midline laparotomy was performed and abdominal aorta and inferior vena cava (IVC) were dissected at the level of iliac bifurcation. Subsequently supratruncal aorta was prepared just below the diaphragm. After the administration of 200 IU/Kg heparin, the perfusion catheter was inserted into the aorta. Renal artery was checked for possible lower pole arteries. Slight mobilization of adrenal gland was done for better exposure of renal vein. The aorta was cross-clamped and the cold perfusion was performed with HTK (histidine tryptophan ketoglutarate) solution (Custodiol®, Dr. F. Köhler Chemie GmbH, Alsbach-Hähnlein, Germany) and the infrarenal IVC was vented. The renal artery was cut without a patch; renal veins were cut with a short IVC cuff. After the procurement, renal artery was catheterized by a soft cannula and perfused again. The kidney was subsequently cold-stored in HTK for 18 h.

### Kidney Transplantation

The details regarding operation procedures have been published elsewhere (7). Briefly, the recipient animals were first premedicated in the same way as the donor animals, anesthetized, ventilated and instrumented. Baseline blood samples were obtained. After a midline laparotomy, the pigs underwent nephrectomy followed by standard kidney transplantation. In summary, right sided kidney transplantation was started with an end-to-side venous anastomosis of the renal vein to IVC with 5-0 Prolene using a continuous suture technique. The arterial anastomosis was performed end-to-side on the aorta in an analogous manner. The kidney was re-perfused first by releasing the venous perfusion by removing the clamp on the vein and, as a second step, releasing the arterial perfusion by removing the clamp on the artery. Subsequently, the ureteroneocystostomy was performed using 5-0 PDS sutures continuously. The two recipient pigs in each recipient group were transplanted simultaneously using two kidneys from each donor pig.

### Post-transplant Procedure

The recipients were monitored on the operating table for 4 h, after which blood samples and protocol biopsies were taken and the abdomen was closed. The indwelling central venous catheter was kept in order to draw blood samples as well as for the intravenous administration of analgesics, antibiotics and volume and substrate substitution. The catheter in the carotid artery was removed after surgery. The animals were then extubated and returned to the cage. Recipients received 0.05 mg/kg buprenorphine, 25–50 mg/kg Metamizole for analgesia as well as 200 mg ciprofloxacin and 125 mg metronidazole, over the remaining central venous catheter. Buprenorphine 0.02–0.05 mg/kg and Metamizole 25–50 mg/kg were given every 12 h for the first 48 h postoperatively.

When the animals were awake and had regained their physiological body temperature, they were taken to the holding area of the University's Interfaculty Biomedical Research Facility. The animals were under observation of the competent animal caretakers and veterinarians, immediately gaining free access to water. On the evening of the operating day, the animals received 500 ml glucose 5% + 500 ml lactated Ringer. On the 1st postoperative day, the animals received 1,000 mL glucose 10% + 1,000 mL ringer lactate. Solid food was allowed only after bowel sound was heard. Parenteral nutrition with Nutriflex peri was considered for animals unable to eat. After the surgery based on pigs' general performance, it was decided how they should be observed and kept. All the pigs were visited three times a day and checked in terms of weight change. Blood was drawn over the central venous catheter daily to measure complete blood count, blood urea nitrogen (BUN), creatinine (Cr) and electrolytes up to postoperative day (POD) 5. No immunosuppression was administered. Animals were sacrificed at the end of the study on POD 5 under deep anesthesia by intravenous injection of potassium chloride (2 mmol/kg).

### Histopathology

To investigate early histopathological changes during kidney transplantation, wedge biopsies were obtained 4 h after reperfusion. Kidney samples were fixed in 10% buffered formalin, routinely embedded in paraffin, cut into 4 μm-thick sections for hematoxylin and eosin stain as well as for Periodic acid-Schiff reaction according to standard protocols. Qualitative assessment of samples was performed to determine and grade acute tubular injury (1 = mild, dilated tubules, partial brush border loss, 2 = moderate, dilated tubules, complete brush border loss, hyaline cylinders, 3 = severe, complete epithelial atrophy, tubule necrosis). Quantitative assessment of acute tubular damage was also performed and scored as quartiles (1 = 0–25, 2 = 26–50, 3 = 51–75, and 4 = 76–100%).

### Immunohistochemistry

For immunohistochemical examination, sections were labeled with commercially available antibodies against cytochrome c (Abcam, Cambridge, UK, ab90529, dilution 1:200) and TNF-α (Abcam, Cambridge, UK, ab6671, dilution 1:50). After heat-induced antigen retrieval at pH 9 (Target Retrieval Solution, Agilent Technologies, Inc., Santa Clara, USA) for cytochrome c and pH 6 (Target Retrieval Solution, Agilent Technologies, Inc., Santa Clara, USA) for TNF-α, respectively, the slides were blocked with Dako REAL Peroxidase-Blocking Solution (Agilent Technologies, Inc., Santa Clara, USA) and incubated with the primary antibody. An anti-rabbit secondary antibody conjugated to HRP (Polyview plus HRP (anti-rabbit) reagent, ENZO Life Sciences GmbH, Lörrach, Germany) was applied. AEC solution (Dako REAL Substrate Solution, Agilent Technologies, Inc., Santa Clara, USA) was used to visualize the signal.

The immunohistochemical scoring was performed according to Allred et al. (8). The Proportion Score (PS) was the estimated percentage ratio of positive TNF-α-stained or cytochrome c-stained cells to the total number of cells, classified as: PS0 (0%), PS1 (>0–1%), PS2 (≥1–10%), PS3 (≥10–33%), PS4 (≥33–66%), and PS5 (≥66–100%). The Intensity Score (IS) was measured based on estimated staining intensity by visual assessment and was scored as: 0 (negative), 1+ (weak), 2+ (moderate), or 3+ (strong). The total score (TS) was calculated as the sum of the PS and IS and ranged from 0 to 8.

### Statistical Analysis

Statistical analysis was performed using IBM SPSS Statistics for Windows, Version 22.0 (IBM Corp. Released 2013. Armonk, NY). Continuous data are expressed as mean values ± standard deviation (SD) and differences between groups were analyzed using the one-way ANOVA test. Categorical data were compared using the chi-square test of association. Histopathological data were analyzed using the Kruskal-Wallis test followed by the Bonferroni *post-hoc* method. *P* < 0.05 were accepted as statistically significant differences.

## Results

**Figure 2:**
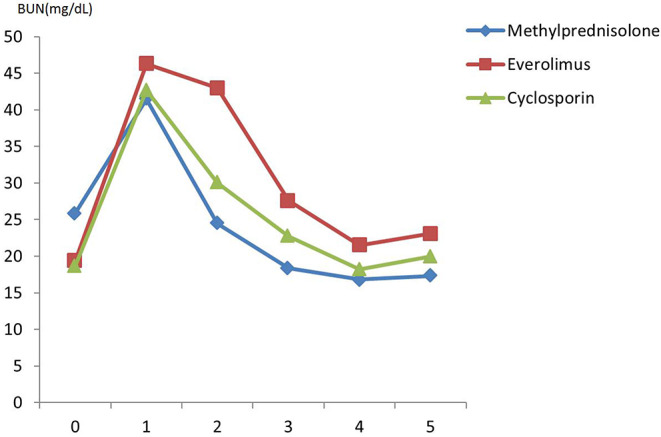
Posttransplant blood urea nitrogen (BUN) in recipients in different study groups.

**Figure 3:**
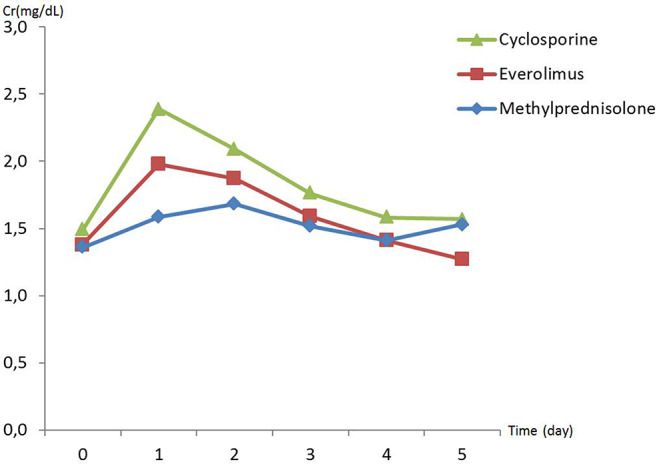
Posttransplant creatinine (Cr) in recipients in different study groups.

**Figure 4:**
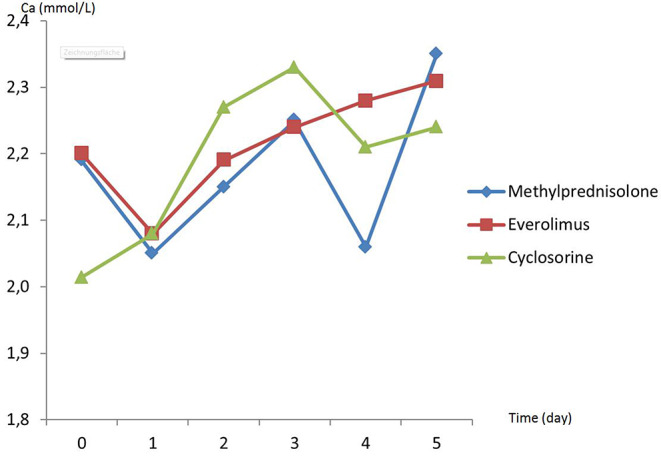
Posttransplant calcium (Ca) in recipients in different study groups.

**Figure 5:**
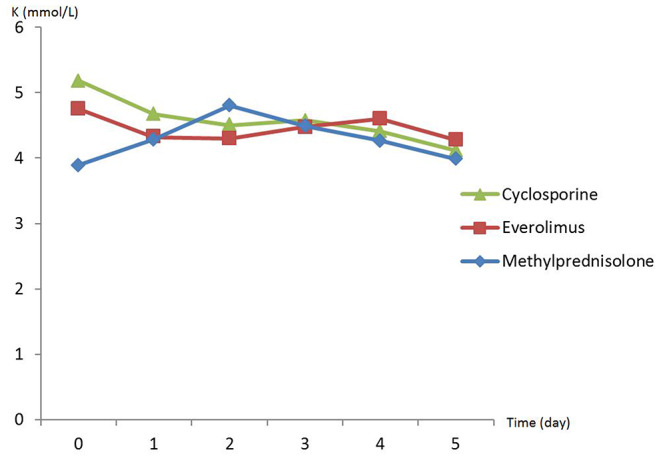
Posttransplant potassium (K) in recipients in different study groups.

There was no difference in preoperative hemodynamic parameters, hemoglobin/hematocrit, electrolytes as well as intraoperative blood loss between the groups (Table 1). The duration of brain death and the ischemia did not vary between the groups, either (Table 2). BUN and Cr increased posttransplant in all groups and returned to normal through POD 4 to 5 (Figures 2, 3) and were not significantly different between the groups except for higher BUN after preconditioning with Everolimus compared to other groups on POD 2 (30 Cyclosporine vs. 43 in Everolimus vs. 24.5 in Methylprednisolone groups, *p* = 0.01) (Figure 2), and higher Cr after preconditioning with Cyclosporine on POD 1 (2.39 in Cyclosporine vs. 1.98 in Everolimus vs. 1.58 in Methylprednisolone, *p* = 0.02) (Figure 3). The electrolytes showed no difference between the groups throughout the study (Figures 4, 5).

**Table 1:** Baseline data.

**Variables**	**Cyclosporine**	**Everolimus**	**Methylprednisolone**	***p* value**
Weight (kg)^*^	32.76 ± 1.88	34.07 ± 2.20	32.25 ± 1.28	0.130
BUN (mg/dl)^*^	18.67 ± 7.45	19.37 ± 5.55	25.87 ± 7.95	0.098
Cr (mg/dl)^*^	1.50 ± 0.34	1.39 ± 0.24	1.36 ± 0.26	0.581
K (mmol/L)^*^	5.19 ± 1.47	4.75 ± 2.14	3.89 ± 0.70	0.243
Ca (mmol/L)^*^	2.01 ± 0.27	2.20 ± 0.17	2.19 ± 0.15	0.122
Hemoglobin (g/dl)^*^	10.38 ± 1.85	11.03 ± 1.47	12.24 ± 1.61	0.087
mean arterial pressure (mmHg)^**^	62.78 ± 3.53	63.67 ± 3.24	65.00 ± 3.50	0.421
heart rate^**^	101.22 ± 4.92	97.56 ± 3.50	99.00 ± 5.26	0.255
Temperature (°C)^**^	35.33 ± 0.22	35.34 ± 0.19	35.47 ± 0.17	0.271
Blood loss (ml)	130 ± 27	137 ± 21	147 ± 17	0.318

*kg, kilogram; mg/dl, milligram per deciliter; mmol/L, millimole per liter; g/dl, gram per deciliter; mmHg, millimeter mercuri; C, centigrade, ml, milliliter; BUN, Blood Urea Nitrogen; Cr, creatinine; K, Kalium; Ca, Calcium*,

* *, preoperative*;

** *, before procurement*.

**Table 2:** Operative times.

	**BD duration [h]**	**CIT duration [h]**	**WIT duration [min]**
Cyclosporine	6.6 ± 2.1	18.4 ± 2.0	48.9 ± 7.7
Everolimus	7.8 ± 0.8	20.9 ± 3.6	48.9 ± 10.5
Methylprednisolone	6.8 ± 1.6	18.5 ± 2.9	43.8 ± 8.3
All groups	7 ± 1.6	19.3 ± 2.9	47.7 ± 8.7
*P*-value	0.39	0.24	0.48

*Data is presented as mean ± SD. BD, brain death; CIT, cold ischemia time; WIT, warm ischemia time; h, hour; min, minute*.

### Histopathological Analysis

Histological assessment revealed no significant differences between the groups (Table 3). A various degree of acute tubular injury was shown in all groups, with a mean score of 1 (<25% tubular damage) in Quantitative assessment, as well as a mean score of 1 (mild tubular injury) in qualitative assessment of acute tubular damage, attributable to post-explant ischemia. A significant difference could neither be shown regarding the severity, nor the quantity of ATI.

**Table 3:** Quantitative and qualitative histopathological assessment of acute tubular injury.

	ATI quantitative^*^	**ATI qualitative^#^**
Cyclosporine	1 (1–2)	1 (1–2)
Everolimus	1 (1–2)	1 (1–2)
Methylprednisolone	1 (1–3)	1 (1–2)
*p* value	0.825	0.491

* *Quantitative assessment of samples was performed to determining acute tubular necrosis as quartiles (1 = 0–25, 2 = 26–50, 3 = 51–75, and 4 = 76–100%)*.

# *Qualitative assessment of samples determined acute tubular injury (ATI) as quartiles (1 = mild, dilated tubules, partial brush border loss, 2 = moderate ATI, dilated tubules, complete brush border loss, hyaline cylinders, 3 = severe ATI, complete epithelial atrophy, tubule necrosis)*.

### Immunohistochemistry

**Figure 6:**
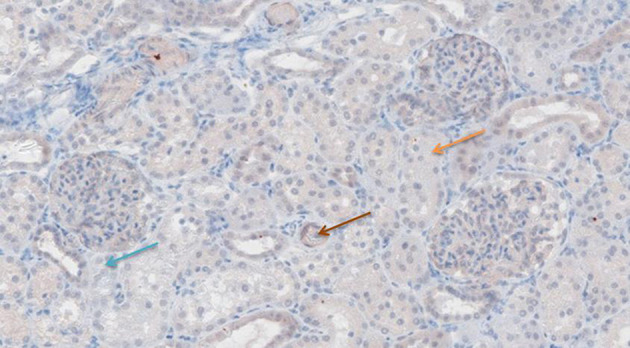
TNF-α antibody staining after preconditioning with Cyclosporine. Arrows show different intensities; blue: intensity 0, orange: intensity 1, brown: intensity 2, and black: intensity 3.

**Figure 7:**
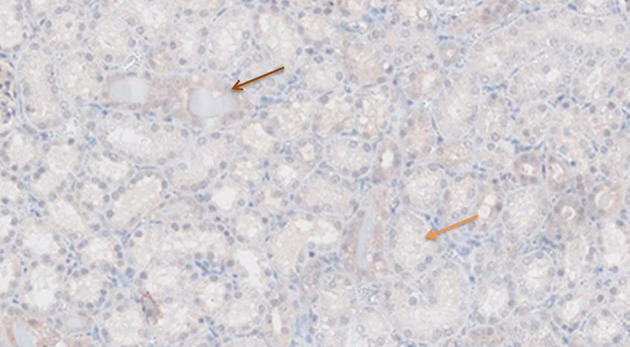
TNF-α antibody staining after preconditioning with Everolimus. Arrows show different intensities; blue: intensity 0, orange: intensity 1, brown: intensity 2, and black: intensity 3.

**Figure 8:**
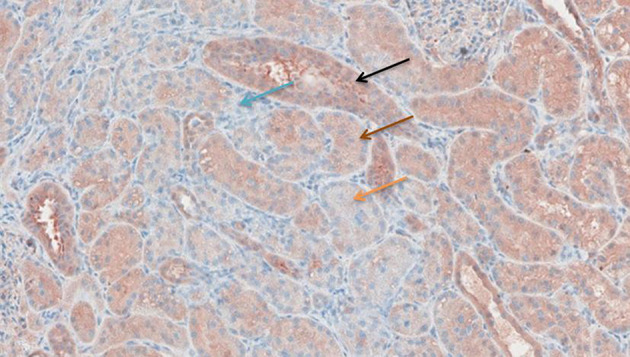
TNF-α antibody staining after preconditioning with Methylprednisolone. Arrows show different intensities; blue: intensity 0, orange: intensity 1, brown: intensity 2, and black: intensity 3.

**Figure 9:**
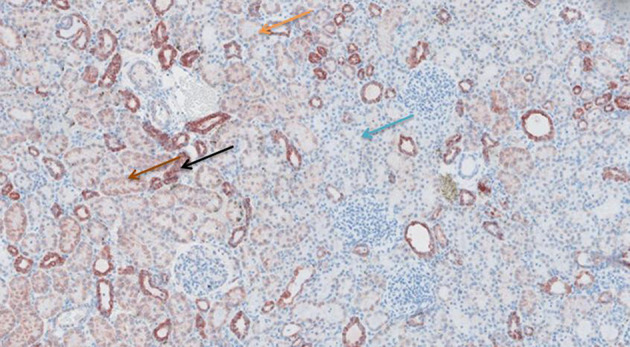
Cytochrome c antibody staining after preconditioning with Cyclosporine. Arrows show different intensities; blue: intensity 0, orange: intensity 1, brown: intensity 2, and black: intensity 3.

**Figure 10:**
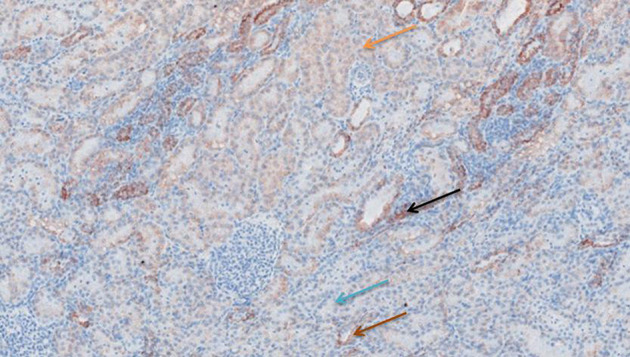
Cytochrome c antibody staining after preconditioning with Everolimus. Arrows show different intensities; blue: intensity 0, orange: intensity 1, brown: intensity 2, and black: intensity 3.

**Figure 11:**
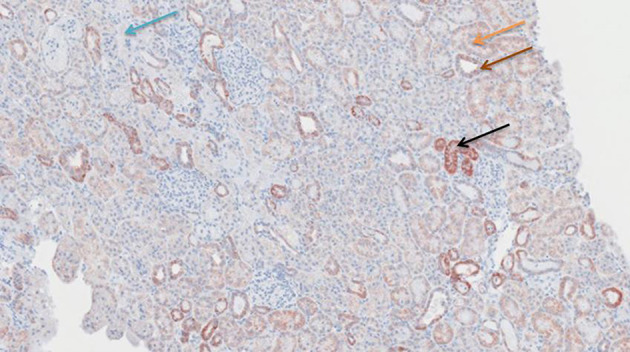
Cytochrome c antibody staining after preconditioning with Methylprednisolone. Arrows show different intensities; blue: intensity 0, orange: intensity 1, brown: intensity 2, and black: intensity 3.

**Figure 12:**
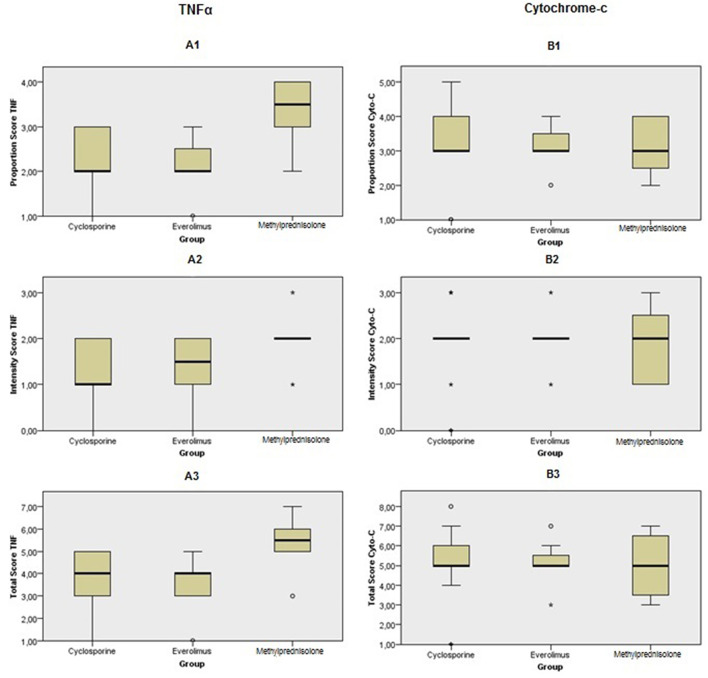
Immunohistochemical scoring after preconditioning with Cyclosporine, Everolimus, and Methylprednisolone 4 h following kidney transplantation. (A1) PS in TNF-α, (A2) IS in TNF-α, (A3) TS in TNF-α, (B1) PS in cytochrome c, (B2) IS in cytochrome c and (B3) TS in cytochrome c. *P* values were calculated for comparison between intervention groups (Everolimus and Cyclosporine) and Methylprednisolone group. PS and TS were significantly different in TNF-α staining between groups. It shows that apoptosis process was started sooner in Methylprednisolone groups rather than the others.

Figures 6–11 show the immunohistochemical staining as well as the (semi)quantitative assessment of the expression of TNF-α and cytochrome c 4 h after reperfusion. TNF-α expression in the immunohistochemistry staining was significantly higher in the Methylprednisolone groups compared with the Everolimus and Cyclosporine groups (*p* = 0.01). This significance was seen in both PS and TS (*P* < 0.01, Figure 12 A1 and A3). There was no difference in cytochrome c expression between the groups.

## Discussion

Brain death triggers an inflammatory response in the donor organs with T lymphocyte and macrophage infiltration and release of multiple proinflammatory cytokines, among all TNF-α, Interleukin-6, and Interleukin-10, which has been shown to enhance the immunogenicity of the organs and potentiate the deleterious effects of IRI after organ transplantation (9). The pharmacologic preconditioning of the donor has been shown to ameliorate the allo-immune response to this enhanced immunogenicity after DBD (10–15). Few studies have investigated pharmacological preconditioning with Cyclosporine in rat kidneys (16, 17). In these studies, preconditioning with Cyclosporine led to improved renal function and histology, increased heat shock protein 70, and decreased expression of pro-inflammatory cytokines (Interleukin-1 and TNF-α) as well as amelioration of oxidative stress after IRI. In contrast, other studies observed aggravated IRI in rat kidney after Cyclosporine, as detected by increased renal dysfunction, decreased Glomerular filtration rate (GFR) and delayed tubular regeneration (18–20). Similarly, there have been reports on negative effects of sirolimus on IRI (including renal dysfunctions, delayed tubular regeneration and increased expression of heme oxygenase-1) (21), while others observed no negative effect of sirolimus pre-treatment on renal outcome after IRI (22). Moreover, data regarding oral donor preconditioning with immunosuppressive agents on the outcome of renal transplantation is scares. One study has shown that the oral donor pharmacological preconditioning with Everolimus or Cyclosporine does not reduce IRI in a rat kidney transplant model (23). We have previously shown that the oral administration of a preconditioning nutritional supplement is protective against IRI in pigs (24). The possible responsible mechanisms include the inactivation of hepatic Kupffer cells via cellular and molecular mechanisms including bacterial translocation and lipopolysaccharide release that prevents the systemic cytokine release, adhesion molecules, leukocyte infiltration and subsequent histological changes. There is a pivotal interaction between the intestinal epithelium, the enteric antigen-presenting cells (e.g., gut dendritic cells), portal circulation, and hepatic kupffer cells, so that the tackling of the IRI via pharmacologic oral preconditioning may significantly modulate the ultimate immune response of the host (25). As for standard application in human kidney transplantation, the absorption phase for CSA occurs over the first 4 h after oral administration. Oral everolimus is absorbed rapidly, and reaches peak concentration after 1.3–1.8 h. For this reason, we administrated the oral CSA and Everolimus only few hours before organ procurement.

To our knowledge, there has been no study on the oral preconditioning of DBD donor in a big animal transplant model. Our present work showed that oral preconditioning with Cyclosporine or Everolimus in DBD pig kidney transplantation is feasible and down-regulates TNF-α expression. The reduction in TNF-α expression seems to be plausible in our model, as an increase of intragraft TNF-α expression is documented in the organs of DBD donors, and after organ reperfusion. TNF-α aggravates the adherence of leukocytes to vascular endothelium leading to enhancement of IRI and acceleration of acute allograft rejection after organ transplantation (26–30). The observed reduction of TNF-α expression might be a hint to suggest an IRI-reducing effect of the preconditioning with Cyclosporine and Everolimus.Cytochrome c, on the other hand, is a hemeprotein in the inner mitochondrial membrane. It has been shown that IRI leads to membrane depolarization by calcium overload in mitochondria, leading to opening of the mitochondrial permeability transition pore. As a result, cytochrome c is released into cytosol and activates the caspase family, leading to apoptosis (31). Our immunohistochemistry stains of cytochrome c showed, however, no difference between the groups. The release of cytochrome c into cytosol might have occured later than 4 h after reperfusion.

In the present work, in order to stimulate the actual clinical practice, we induced hypotensive brain death in our donors, and allowed 6 h' time for the inflammatory response following brain death to develop. Moreover, we kept an average of 18 h cold ischemia time to enhance IRI. No posttransplant immunosuppression therapy in recipients was administered to avoid confounding bias.

In order to detect the early allograft changes after IRI, the protocol biopsies were performed 4 h after reperfusion. Kusaka et al. have shown that the early changes including the expression of the inflammatory proteins could take place as early as 1 h after the implantation of the kidneys after DBD (32). Although we could show a difference in the expression of TNF-α between the study groups after four hours, our conventional histopathological studies were not different between the different groups, which might imply that the interval being too short to observe the histological changes of tubular damage. Although we have followed the recipients until POD 5 for the quality controlling of the transplants, we did not look for POD 5 biopsies as the findings would have not been specifically attributable to the immunological effects after DBD, and rather acute rejection. This was to avoid the acute rejection as a confounding bias. The clinical as well as lab data through POD 5 including the laboratory tests showed no relevant difference between the groups.

The present work has its own limitations. Although we administered the routine immunosuppressive doses, data on the appropriate oral doses of Cyclosporine and Everolimus for the purpose of oral preconditioning is lacking. Furthermore, the best time points for the administration of the oral preconditioning agents as well as the frequency of medication are not known and vary widely among different studies. Furthermore, the best time point to look for the early innate host immune response triggered synergistically by IRI and DBD in allografts is still unclear.

In summary, our findings suggest the feasibility of the oral preconditioning with CNI or mTORi in DBD donors in pig kidney transplantation. A reduced expression of TNF-α in transplanted organs in the early post-transplant phase was seen after oral preconditioning with these agents. Our data can serve as a platform for future experimental and clinical studies to evaluate the protecting role of donor oral preconditioning against IRI and its clinical relevance.

## Data Availability Statement

All datasets generated for this study are included in the article/supplementary material.

## Ethics Statement

The animal study was reviewed and approved by Regierungspräsidium Karlsruhe, Baden-Württemberg, Germany (file number: 35-9185.81/G-5/16).

## Author Contributions

AN, AMe, and SA developed the original concept of the study. AN, SA, MN, OG, and EK developed the design and methodology. SA, OG, EK, MN, AA, SM, AMa, MS, MK, and AY participated in the operations and data collection. CE and TP participated in the pathological assessment. MN, EK, OG, and AA performed the statistical assessments and developed the analysis plan. SA, AN, MN, EK, OG, and AA contributed to drafting the article. AN, NG, MG, SM, AMa, MS, MK, AY, and AMe contributed to the revision of the final report. All authors read and approved the final manuscript.

## Conflict of Interest

The authors declare that the research was conducted in the absence of any commercial or financial relationships that could be construed as a potential conflict of interest.

## Abbreviations

DBD: donors after brain death
IRI: ischemia-reperfusion injury
CNIs: calcineurin inhibitors
mTORI: inhibitors of mammalian target for Rapamycin
HTK: Histidine-Tryptophane-Ketoglutarate
TNF-α: tumor necrosis factor alpha
IL-6: interleukin 6
IL-10: interleukin 10
GFR: Glomerular filtration rate
POD: post-operative day
SD: Standard deviation
i.m.: intramuscularly
i.v.: intravenously
MAP: mean arterial pressure
CVP: central venous pressure
HR: heart rate
IVC: inferior vena cava
PS: proportion score
IS: intensity score
TS: total score
BUN: blood urea nitrogen
Cr: creatinine
ATI: acute tubular injury.

## References

[1] De Vries D Lindeman J Ringers J Reinders M Rabelink T Schaapherder A. Donor brain death predisposes human kidney grafts to a proinflammatory reaction after transplantation. Am J Transplant. (2011) 11:1064–70. 10.1111/j.1600-6143.2011.03466.x 21449948

[2] Pratschke J Wilhelm MJ Kusaka M Basker M Cooper DK Hancock WW. Brain death and its influence on donor organ quality and outcome after transplantation. Transplantation. (1999) 67:343–8. 10.1097/00007890-199902150-00001 10030276

[3] Kotsch K Ulrich F Reutzel-Selke A Pascher A Faber W Warnick P. Methylprednisolone therapy in deceased donors reduces inflammation in the donor liver and improves outcome after liver transplantation: a prospective randomized controlled trial. Ann Surg. (2008) 248:1042–50. 10.1097/SLA.0b013e318190e70c 19092349

[4] Shihab FS Bennett WM Andoh TF. Donor preconditioning with a calcineurin inhibitor improves outcome in rat syngeneic kidney transplantation. Transplantation. (2009) 87:326–9. 10.1097/TP.0b013e3181945332 19202436

[5] Golling M Mehrabi A Blum K Jahnke C Kellner H Bud O. Effects of hemodynamic instability on brain death-induced prepreservation liver damage1. Transplantation. (2003) 75:1154–9. 10.1097/01.TP.0000062868.34247.8F 12717195

[6] Golling M Jahnke C Fonouni H Ahmadi R Urbaschek R Breitkreutz R. Distinct effects of surgical denervation on hepatic perfusion, bowel ischemia, and oxidative stress in brain dead and living donor porcine models. Liver Transpl. (2007) 13:607–17. 10.1002/lt.21069 17394167

[7] Golriz M Fonouni H Nickkholgh A Hafezi M Garoussi C Mehrabi A. Pig kidney transplantation: an up-to-date guideline. Eur Surg Res. (2012) 49:121–9. 10.1159/000343132 23172014

[8] Allred DC Harvey JM Berardo M Clark GM. Prognostic and predictive factors in breast cancer by immunohistochemical analysis. Mod Pathol. (1998) 11:155–68. 9504686

[9] Weiss S Kotsch K Francuski M Reutzel-Selke A Mantouvalou L Klemz R. Brain death activates donor organs and is associated with a worse I/R injury after liver transplantation. Am J Transplant. (2007) 7:1584–93. 10.1111/j.1600-6143.2007.01799.x 17430397

[10] Ryan JB Hicks M Cropper JR Garlick SR Kesteven SH Wilson MK Sodium-hydrogen exchanger inhibition, pharmacologic ischemic preconditioning, or both for extended cardiac allograft preservation. Transplantation. (2003) 76:766–71. 10.1097/01.Tp.0000079254.81264.6d 14501850

[11] van der Woude FJ Schnuelle P Yard BA. Preconditioning strategies to limit graft immunogenicity and cold ischemic organ injury. J Investig Med. (2004) 52:323–9. 10.1136/jim-52-05-32 15551655

[12] Guan X Dei-Anane G Liang R Gross ML Nickkholgh A Kern M. Donor preconditioning with taurine protects kidney grafts from injury after experimental transplantation. J Surg Res. (2008) 146:127–34. 10.1016/j.jss.2007.06.014 18061615

[13] Cicora F Stringa P Guerrieri D Vasquez D Toniolo F Roberti J. Evaluation of histological damage of solid organs after donor preconditioning with thymoglobulin in an experimental rat model. Transpl Immunol. (2013) 28:203–5. 10.1016/j.trim.2013.04.002 23597700

[14] Spindler RS Schnuelle P Nickels L Jarczyk J Waldherr R Theisinger S. N-octanoyl dopamine for donor treatment in a brain-death model of kidney and heart transplantation. Transplantation. (2015) 99:935–41. 10.1097/tp.0000000000000577 25675202

[15] Li S Korkmaz-Icoz S Radovits T Ruppert M Spindler R Loganathan S. Donor preconditioning after the onset of brain death with dopamine derivate n-octanoyl dopamine improves early posttransplant graft function in the rat. Am J Transplant. (2017) 17:1802–12. 10.1111/ajt.14207 28117941

[16] Yang CW Ahn HJ Han HJ Kim WY Li C Shin MJ. Pharmacological preconditioning with low-dose cyclosporine or Fk506 reduces subsequent ischemia/reperfusion injury in rat kidney1, 2. Transplantation. (2001) 72:1753–9. 10.1097/00007890-200112150-00008 11740384

[17] Singh D Chander V Chopra K. Cyclosporine protects against ischemia/reperfusion injury in rat kidneys. Toxicology. (2005) 207:339–47. 10.1016/j.tox.2004.09.018 15664262

[18] Delbridge MS Shrestha BM Raftery AT El Nahas A Haylor J. FTY720 reduces extracellular matrix expansion associated with ischemia-reperfusion induced injury. Transplant Proc. (2007) 39:2992–6. 10.1016/j.transproceed.2007.04.027 18089307

[19] Delbridge MS Shrestha BM Raftery AT El Nahas AM Haylor JL. Reduction of ischemia-reperfusion injury in the rat kidney by FTY720, a synthetic derivative of sphingosine. Transplantation. (2007) 84:187–95. 10.1097/01.tp.0000269794.74990.da 17667810

[20] Goncalves G Cenedeze M Feitoza C de Paula C Marques G Pinheiro H. The role of immunosuppressive drugs in aggravating renal ischemia and reperfusion injury. Transplant Proc. (2007) 39:417–20. 10.1016/j.transproceed.2007.01.027 17362745

[21] Goncalves G Cenedeze MA Feitoza C Wang P Bertocchi A Damiao M. The role of heme oxygenase 1 in rapamycin-induced renal dysfunction after ischemia and reperfusion injury. Kidney Int. (2006) 70:1742–9. 10.1038/sj.ki.5001893 17003813

[22] Inman SR Davis NA Olson KM Lukaszek VA McKinley MR Seminerio JL. Rapamycin preserves renal function compared with cyclosporine A after ischemia/reperfusion injury. Urology. (2003) 62:750–4. 10.1016/s0090-4295(03)00475-8 14550466

[23] Martinez-Palli G Hirose R Liu T Xu F Dang K Feiner J Donor pre-treatment with everolimus or cyclosporine does not reduce ischaemia–reperfusion injury in a rat kidney transplant model. Nephrol Dial Transplant. (2010) 26:1813–20. 10.1093/ndt/gfq646 21068143

[24] Nickkholgh A Li Z Yi X Mohr E Liang R Mikalauskas S. Effects of a preconditioning oral nutritional supplement on pig livers after warm ischemia. HPB Surg. (2012) 2012:783479. 10.1155/2012/783479 22791934 PMC3389686

[25] Yoneyama H Ichida T. Recruitment of dendritic cells to pathological niches in inflamed liver. Med Mol Morphol. (2005) 38:136–41. 10.1007/s00795-005-0289-0 16170461

[26] Amado JA Lopez-Espadas F Vazquez-Barquero A Salas E Riancho JA Lopez-Cordovilla JJ. Blood levels of cytokines in brain-dead patients: relationship with circulating hormones and acute-phase reactants. Metabolism. (1995) 44:812–6. 10.1016/0026-0495(95)90198-1 7540249

[27] Takada M Nadeau KC Hancock WW Mackenzie HS Shaw GD Waaga AM. Effects of explosive brain death on cytokine activation of peripheral organs in the rat. Transplantation. (1998) 65:1533–42. 10.1097/00007890-199806270-00001 9665067

[28] Wilhelm MJ Pratschke J Beato F Taal M Kusaka M Hancock WW. Activation of the heart by donor brain death accelerates acute rejection after transplantation. Circulation. (2000) 102:2426–33. 10.1161/01.cir.102.19.2426 11067799

[29] Pratschke J Kofla G Wilhelm MJ Vergopoulos A Laskowski I Shaw GD. Improvements in early behavior of rat kidney allografts after treatment of the brain-dead donor. Ann Surg. (2001) 234:732–40. 10.1097/00000658-200112000-00004 11729379 PMC1422132

[30] Smith M. Physiologic changes during brain stem death–lessons for management of the organ donor. J Heart Lung Transplant. (2004) 23:S217–222. 10.1016/j.healun.2004.06.017 15381167

[31] Waterhouse NJ Trapani JA. A new quantitative assay for cytochrome c release in apoptotic cells. Cell Death Differ. (2003) 10:853–5. 10.1038/sj.cdd.4401263 12815469

[32] Kusaka M Kuroyanagi Y Kowa H Nagaoka K Mori T Yamada K. Genomewide expression profiles of rat model renal isografts from brain dead donors. Transplantation. (2007) 83:62–70. 10.1097/01.tp.0000250485.53865.b8 17220792

